# RNA sequencing analysis reveals increased expression of interferon signaling genes and dysregulation of bone metabolism affecting pathways in the whole blood of patients with osteogenesis imperfecta

**DOI:** 10.1186/s12920-020-00825-7

**Published:** 2020-11-23

**Authors:** Lidiia Zhytnik, Katre Maasalu, Ene Reimann, Aare Märtson, Sulev Kõks

**Affiliations:** 1 Clinic of Traumatology and Orthopedics, Tartu University Hospital, Puusepa 8, 51014 Tartu, Estonia; 2 Department of Traumatology and Orthopedics, Institute of Clinical Medicine, University of Tartu, Tartu, Estonia; 3 Estonian Genome Centre, University of Tartu, Tartu, Estonia; 4 Perron Institute for Neurological and Translational Science, QEII Medical Centre, Nedlands, WA Australia; 5 Centre for Molecular Medicine and Innovative Therapeutics, Murdoch University, Murdoch, WA Australia

**Keywords:** Osteogenesis imperfecta, Bone fragility, Transcriptome, RNA-seq, Inflammation, Bone metabolism

## Abstract

**Background:**

Osteogenesis imperfecta (OI) is a rare genetic disorder in which the patients suffer from numerous fractures, skeletal deformities and bluish sclera. The disorder ranges from a mild form to severe and lethal cases. The main objective of this pilot study was to compare the blood transcriptional landscape of OI patients with *COL1A1* pathogenic variants and their healthy relatives, in order to find out different gene expression and dysregulated molecular pathways in OI.

**Methods:**

We performed RNA sequencing analysis of whole blood in seven individuals affected with different OI severity and their five unaffected relatives from the three families. The data was analyzed using edgeR package of R Bioconductor. Functional profiling and pathway analysis of the identified differently expressed genes was performed with g:GOSt and MinePath web-based tools.

**Results:**

We identified 114 differently expressed genes. The expression of 79 genes was up-regulated, while 35 genes were down-regulated. The functional analysis identified a presence of dysregulated interferon signaling pathways (*IFI27, IFITM3, RSAD12, GBP7*). Additionally, the expressions of the genes related to extracellular matrix organization, Wnt signaling, vitamin D metabolism and MAPK-ERK 1/2 pathways were also altered.

**Conclusions:**

The current pilot study successfully captured the differential expression of inflammation and bone metabolism pathways in OI patients. This work can contribute to future research of transcriptional bloodomics in OI. Transcriptional bloodomics has a strong potential to become a major contributor to the understanding of OI pathological mechanisms, the discovery of phenotype modifying factors, and the identification of new therapeutic targets. However, further studies in bigger cohorts of OI patients are needed to confirm the findings of the current work.

## Background

Osteogenesis Imperfecta (OI) is a rare bone fragility disorder. The main features or characteristics of this genetic disorder are multiple bone fractures, skeletal deformities, bluish sclera and short stature. Additionally, some patients may develop dentinogenesis imperfecta (DI), hearing loss, cardiovascular and pulmonary complications, underlining the complex nature of the disorder [1].

OI is classified into five clinical types: type I is the most common, being a mild OI with bluish sclera; type II is a perinatally lethal OI, type III is a progressively deforming OI; type IV is a variable OI with white sclera; and type V is an OI with abnormal mineralization. Genetic OI classification, however, has 20 OI types, which is based on the identified molecular mechanisms of the disease. Correlations between OI genotype and phenotype are elusive, and high phenotypic variability between the affected individuals with the same variant is present [2–4].

Around 90% of all OI cases are associated with collagen type I structural or amount defects because of *COL1A1* and *COL1A2* pathogenic variants [5]. Being the main structural protein in the human body, collagen type I is the main organic component of the bone [6]. An abnormal or reduced amount of collagen type I affects bone metabolism in numerous ways. Bone tissue in OI patients was described with high bone turnover, hypermineralization, defective extracellular matrix (ECM), hypercellular structure and apoptosis of bone cells [7–9]. In recent years, more and more attention is being paid to the inflammatory component in OI pathogenesis. Elevated pro-inflammatory cytokine levels were reported in a murine model of OI (TGF-β, TNF-α), as well as in OI children (platelet counts) [10–12]. Moreover, targeting the inflammation is predicted to be a novel therapeutic approach for OI [10]. At present, the therapies influencing TGF-β and Wnt pathways are broadly tested, as no cure is available for OI and a search of effective therapies for this genetic disorder continues [13, 14].

In their search for new pathological pathways, biomarkers and therapies, many investigators use whole genome RNA sequencing (RNAseq) [15]. The approach is sensitive, robust and powerful, and is designed to discover differently expressed genes (DEGs) and pathways, and is gaining more popularity in research of monogenic diseases, mainly for diagnostics [16]. However, transcriptome analysis is much more promising, allowing identification of drug-gene interactions and genes modification [17–19].

Although the main affected tissue in OI is bone, obtaining bone biopsies is an invasive procedure and hard to access; this is where transcriptional bloodomics comes in. Blood transcriptome was proposed as an alternative for diseases-specific molecular profiles because it shares ~ 80% of transcriptome with major tissues and reflects the functional state of cells, gene regulation and inflammatory response [20–22].

The main aim of the present study was to identify DEGs and dysregulated pathways in the whole blood of OI patients with *COL1A1* pathogenic variants, in order to examine the validity of blood RNAseq for capturing OI-related pathological mechanisms.

## Methods

### Patients and controls

**Fig. 1:**
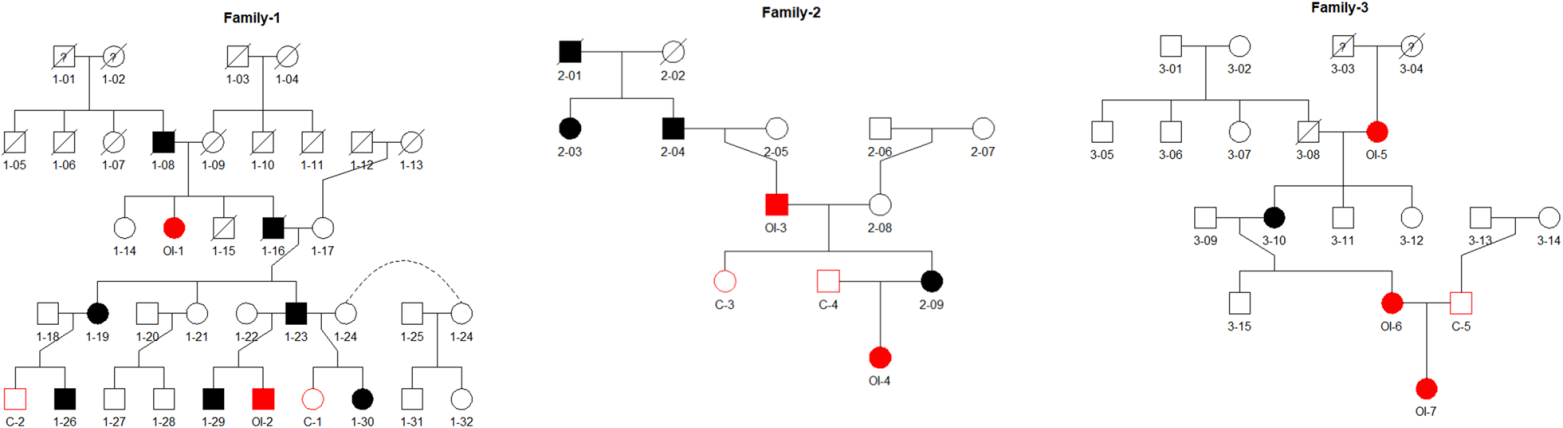
Pedigree trees of three Estonian OI families, which participated in the study. The males are pictured as squares and females as circles. Deceased family members are defined with a cross line. Affected individuals are represented with colored-in symbols. Individuals that were recruited in the study are depicted in red color

A total of 12 individuals from the three OI families, of Estonian origin, were enrolled in the study (Fig. 1). Seven individuals were affected by OI (OI1-7) and five unaffected individuals from the same families (C1-5) were treated as healthy controls. The included families were selected from the Osteogenesis Imperfecta database (UT OI database) of the Clinic of Traumatology and Orthopedics, University of Tartu (Estonia). The characteristics of the patients and the controls (lock time 2013) are summarized in Table 1.

**Table 1:** Characteristics of OI patients and normal controls (lock time 2013)

Sample ID	Family no	Sex	Age	Genotype	OI type
OI-1	Family-1	Female	76	*COL1A1*, c.1821 + 1G > A	III
OI-2	Family-1	Male	19	*COL1A1*, c.1821 + 1G > A	I
OI-3	Family-2	Male	46	*COL1A1,* c.750 + 2 T > A	III
OI-4	Family-2	Female	4	*COL1A1,* c.750 + 2 T > A	IV
OI-5	Family-3	Female	75	*COL1A1,* c.1128delT (p.Gly377Alafs*164)	III
OI-6	Family-3	Female	26	*COL1A1,* c.1128delT (p.Gly377Alafs*164)	IV
OI-7	Family-3	Female	2	*COL1A1,* c.1128delT (p.Gly377Alafs*164)	I
C-1	Family-1	Female	26	–	Healthy
C-2	Family-1	Male	26	–	Healthy
C-3	Family-2	Female	12	–	Healthy
C-4	Family-2	Male	28	–	Healthy
C-5	Family-3	Male	31	–	Healthy

#### Family-1

Family-1 harbored a heterozygous *COL1A1* c.1821 + 1G > A splice site pathogenic variant. Patient OI-1 was a 76-year-old female who suffered from OI type III. She had a large number of fractures, major skeletal deformities, including severe kyphoscoliosis, reduced height, triangular face and severe osteoporosis. The patient also had blue sclera and hearing loss. Patient OI-2 was a 19-year-old grandnephew of OI-1 patient. He had OI type I with five fractures, light skeletal deformities, normal height, bluish sclera and no hearing loss so far. C-1 was a healthy half-sister and C-2 was a healthy cousin of patient OI-2.

#### Family-2

Family-2 had a heterozygous *COL1A1* c.750 + 2T> A splice site OI-causative variant. Patient OI-3 was a 46-year-old male with OI type III. He had a total number of 50 diagnosed fractures, had bluish sclera and no hearing loss, and had been a wheelchair user since early childhood. His four-year-old affected granddaughter, patient OI-4, had OI type IV, with a total of four fractures. She had no DI or hearing loss. C-3 and C-4 were an unaffected aunt and father of patient OI-4.

#### Family-3

Family-3 carried a heterozygous *COL1A1* c.1128_delT (p.Gly337Alafs*164) frameshift variant. Patient OI-5 was a 75-year-old woman with OI type III. She had developed severe OI with plenty of fractures (n = 30), long bones deformities, scoliosis, body disproportions, and significantly reduced height. The patient had also developed mild hearing loss, which might be related to her advanced age, although the etiology of the hearing loss was unclear. Her 26-year-old granddaughter, patient OI-6, had OI type IV, with up to 20 bone fractures and normal height. Patient OI-7 was a 2-year-old daughter of patient OI-6 who had OI type I and no fractures until 2013, and was of normal height. The last follow-up confirmed the emergence of two fractures between 2013 and 2020. All the affected individuals in the family had blue sclera, and lacked DI. In addition, patients OI-6 and OI-7 did not suffer from hearing loss. C-5 was a healthy unaffected father of patient OI-7.

### RNA extraction and quality assessment

Whole blood was collected in Tempus Blood RNA Tubes (Applied Biosystems, Life Technologies Corp., Carlsbad, CA, USA) from the OI patients and healthy controls. Further processing and total RNA extraction was performed with a commercial Tempus Spin RNA Isolation Kit (Ambion, Life Technologies Corp., Carlsbad, CA, USA) and RNA purification was performed with GLOBINclear™ Kit (Ambion, Life Technologies Corp., Carlsbad, CA, USA) following the protocols described previously by Maasalu et al. [23]. The quality of total RNA was assessed with Agilent 2100 Bioanalyzer and RNA 6000 Nano kit (Agilent Technologies Inc., Santa Clara, CA, USA).

### RNA-seq library construction and RNA sequencing

RNA-seq library construction and RNA sequencing analysis were performed with a SOLiD 5500 W platform (Life Technologies Corp., Carlsbad, CA, USA) as described by Maasalu et al. [23].

### Sequence reads mapping

Raw reads were processed and mapped using Lifescope 2.5.1 software (Life Technologies Corp., Carlsbad, CA, USA). This whole transcriptome analysis workflow generated output, which includes gene and exon counts, alternative splicing, and fusion transcripts. Raw read counts per gene were input for further statistical analysis.

### Differential gene expression analysis

Differential expression analysis was conducted using R Bioconductor package edgeR (Empirical Analysis of Digital gene Expression Data in R, version 3.28.0) standard workflow with exact test. The edgeR package implements exact statistical methods and generalized linear models for multi-group and multifactorial experiments [24]. One feature of the edgeR approach is an empirical Bayes method that permits the estimation of gene-specific biological variation, even for experiments with minimal levels of biological replication. For the current study we applied model-based normalization and used a negative binomial model. Testing for differential expression was done with the exact test. Statistically significant genes were indicated with adjusted *p *value < 0.05 and false discovery rate (FDR) < 0.05. To overcome issues of biological variability estimation in the presence of minimal number of biological replicates, we used an analysis of fold changes. Logarithmic fold change (logFC) > 1.5 or < −1.5 was used as an additional cut-off for differential gene expression.

Heatmap clustering analysis was generated with the mixOmics package in R (Omics Data Integration Project, version 6.10.6) [25].

### Functional and pathway analysis

Gene pathway analysis was generated with g:Profiler’s g:GOSt functional profiler tool (https://biit.cs.ut.ee/gprofiler/gost), a web server for functional enrichment analysis and conversions of gene lists [26]. Statistical significance was based on a Benjamini–Hochberg FDR approach, used to count multiple comparisons in the analysis, with a threshold less than 0.05. Annotation data sets for the analysis included Reactome, Kyoto Encyclopedia of Genes and Genomes (KEGG) and WikiPathways.

Additionally, RNAseq data was analyzed with a web-based MinePath analysis tool (http://minepath.org/) for the identification of dysregulated functional pathways [27, 28]. MinePath analysis is based on the identification of differently expressed functional pathways within a gene regulatory network using gene expression data analysis. The method is based on the identification of the phenotype differential sub-paths in molecular pathways.

## Results

### Differential gene expression

A total number of 114 genes were differently expressed (with FDR < 0.05) in the whole blood of OI patients and healthy control group (Additional file 1). Out of these, 79 genes were up-regulated and 35 genes were down-regulated. A total of 39 genes which had logFC > 1.5 or < −1.5 were considered significant DEGs (Table 2). Out of the 39 significant DEGs, 31 DEGs were up-regulated and 8 were down-regulated.

**Table 2:** Fifteen differently expressed genes in whole blood of OI patients

Gene symbol	Gene name	Specific function	logFC	*p* value	FDR
*IFI27*	Interferon, alpha-inducible protein 27	Adaptor protein involved into IFN type-I -induced apoptosis	3.309	1.70E−29	4.00E−25
*MTRNR2L1*	MT-RNR2-like 1	Neuroprotective, antiapoptotic factor	2.241	3.50E−28	4.00E−24
*RAP1GAP*	RAP1 GTPase activating protein	GTPase activator	2.825	3.30E−21	3.00E−17
*ADGRG7*	Adhesion G Protein-Coupled Receptor G7	Transmembrane signaling	5.601	4.00E−19	2.00E−15
*RSAD2*	Radical S-adenosyl methionine domain containing 2	Antiviral state induced with IFN type I and II	1.721	1.90E−17	9.00E−14
*IFI44L*	Interferon-induced protein 44-like	Antiviral activity	1.724	6.70E−17	3.00E−13
*LTF*	Lactoferrin (Lactotransferrin)	Iron binding protein. Anabolic, differentiating and anti-apoptotic effects on osteoblasts, inhibits osteoclastogenesis, possibly playing a role in the regulation of bone growth	1.684	2.00E−15	7.00E−12
*JCHAIN*	Immunoglobulin J polypeptide, linker protein for immunoglobulin alpha and mu polypeptides	Serves to link two monomer units of either IgM or IgA	1.576	2.80E−15	9.00E−12
*ALAS2*	Aminolevulinate, delta-, synthase 2	Enzyme, participates in Gly, Ser, Thr metabolism	1.541	5.10E−15	1.00E−11
*IFITM3*	Interferon-induced transmembrane protein 3	Antiviral protein, involved in vacuolar ATPase structural stability	1.578	2.00E−14	5.00E−11
*RNF182*	Ring finger protein 182	E3 ubiquitin-protein ligase that mediates the ubiquitination of ATP6V0C	1.658	3.20E−14	7.00E−11
*MMP8*	Matrix metallopeptidase 8 (neutrophil collagenase)	Cleavage of interstitial collagens in the triple helical domain, collagen type I degradation	2.023	3.50E−14	7.00E−11
*DEFA3*	Defensin Alpha 3	Antimicrobial and cytotoxic peptide involved in host defense	2.027	1.10E−11	2.00E−08
*DEFA1B_dup3*	-	-	2.027	1.10E−11	2.00E−08
*DEFA1_dup3*	-	-	2.027	1.10E−11	2.00E−08
*OLFM4*	Olfactomedin 4	In myeloid leukemia cell lines, inhibits cell growth and induces cell differentiation and apoptosis	1.93	4.50E−10	5.00E−07
*BCORP1*	BCL6 corepressor pseudogene 1	Pseudogene	−1.531	7.20E−08	5.00E−05
*RIMBP2*	RIMS binding protein 2	Plays a role in the synaptic transmission as bifunctional linker	3.849	8.20E−07	5.00E−04
*PNMA8A*	Paraneoplastic Ma antigen family-like 1	Gene associated with oligodendroglioma	3.099	1.90E−06	1.00E−03
*CELA1*	Chymotrypsin-like elastase family, member 1	Hydrolysis of proteins, including elastin	−1.536	2.50E−06	1.00E−03
*SHCBP1*	SHC SH2-domain binding protein 1	Testis-specific spindle-associated factor that plays a role in spermatogenesis	1.554	5.20E−06	2.00E−03
*ZC3HAV1L*	Zinc finger CCCH-type, antiviral 1-like	Antiviral protein	2.473	1.00E−05	4.00E−03
*UBE2C*	Ubiquitin-conjugating enzyme E2C	Accepts ubiquitin from the E1 complex and catalyzes its covalent attachment to other proteins	1.587	1.30E−05	5.00E−03
*NEK8*	NIMA-related kinase 8	Required for renal tubular integrity. May regulate local cytoskeletal structure in kidney tubule epithelial cells	1.676	1.40E−05	5.00E−03
*SDC1*	Syndecan 1	Cell surface proteoglycan that bears both heparan sulfate and chondroitin sulfate and that links the cytoskeleton to the interstitial matrix	4.825	1.60E−05	6.00E−03
*CEACAM6*	Carcinoembryonic antigen-related cell adhesion molecule 6 (non-specific cross reacting antigen)	Cell surface glycoprotein that plays a role in cell adhesion and tumor progression	1.595	1.80E−05	6.00E−03
*TCL6*	T-cell leukemia/lymphoma 6 (non-protein coding)	RNA Gene, and is affiliated with the misc_RNA class. May have a function in neuronal differentiation and/or axon growth	1.788	2.80E−05	9.00E−03
*ZBTB32*	Zinc finger and BTB domain containing 32	DNA-binding protein. May function as a transcriptional transactivator and transcriptional repressor	2.015	2.90E−05	9.00E−03
*GBP7*	Guanylate binding protein 7	GTP to GMP hydrolysis	1.541	3.20E−05	1.00E−02
*STOX1*	Storkhead box 1	Involved in regulating the levels of reactive oxidative species and reactive nitrogen species	−1.856	5.30E−05	1.60E−02
*KCNN3*	Potassium intermediate/small conductance calcium-activated channel, subfamily N, member 3	Forms a voltage-independent potassium channel activated by intracellular calcium	4.615	7.00E−05	2.00E−02
*TM4SF19*	Transmembrane 4 L six family member 19	Members of this family function in various cellular processes, including cell proliferation, motility, and adhesion via their interactions with integrins	−1.632	9.70E−05	2.60E−02
*DKK3*	Dickkopf Wnt signaling pathway inhibitor 3	Antagonizes canonical Wnt signaling by inhibiting LRP5/6 interaction with Wnt	−1.57	9.90E−05	2.60E−02
*GLDC*	Glycine dehydrogenase (decarboxylating)	The glycine cleavage system catalyzes the degradation of glycine	2.858	1.00E−04	2.70E−02
*LOC400931*	–	–	−1.978	1.40E−04	3.30E−02
*GFPT2*	Glutamine-fructose-6-phosphate transaminase 2	Controls the flux of glucose into the hexosamine pathway	−2.417	1.40E−04	3.40E−02
*MDGA1*	MAM domain containing glycosylphosphatidylinositol anchor 1	Plays a role in the formation or maintenance of inhibitory synapses	1.599	1.60E−04	3.80E−02
*G0S2*	G0/G1 switch 2	Promotes apoptosis	1.555	1.80E−04	4.20E−02
*SLC1A7*	Solute carrier family 1 (glutamate transporter), member 7	Transports L-glutamate	−1.743	2.20E−04	4.70E−02

*logFC* logarithmic fold change, *FDR* false discovery rate

The five most significant DEGs which were up-regulated in the OI samples were interferon, alpha-inducible protein 27 (*IFI27,* *p* value 1.73E−29, FDR 4.21E−25)*,* MT-RNR2-like 1 (*MTRNR2L1,* *p* value 3.49E−28, FDR 4.25E−24)*,* RAP1 GTP-ase activating protein *(RAP1GAP,* *p* value 3.26E−21, FDR 2.65E−17)*,* Adhesion G Protein-Coupled Receptor G7 (*ADGRG7,* *p* value 4.01E−19, FDR 2.44E−15) and Radical S-adenosyl methionine domain containing 2 (*RSAD2,* *p* value 1.91E−17, FDR 9.31E−14) genes.

**Fig. 2:**
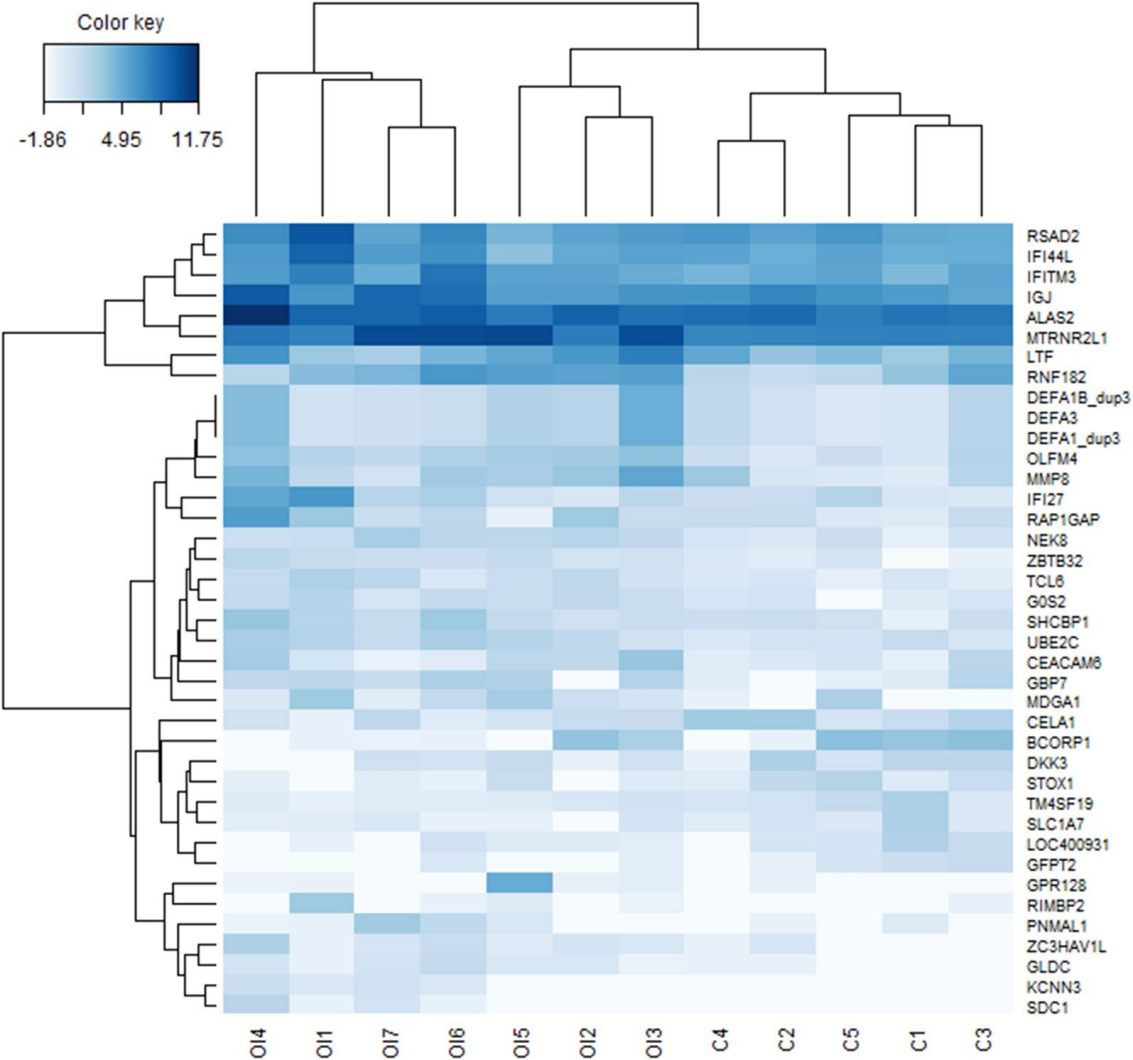
Heatmap of the 39 significant DEGs, illustrating differences in expressional profiles between OI patients (OI1-7) and healthy controls (C1-5)

Statistically significant DEGs were clustered with Heatmap analysis (Fig. 2). In this figure, the horizontal axis represents OI patients (OI1-7) and healthy control (C1-5) groups, whereas vertical axis represents the expression of all 39 identified DEGs. The Heatmap enabled us to see clustering of DEGs expression between samples from the cohort of OI patients and healthy controls.

### Pathway and network analysis

**Fig. 3:**
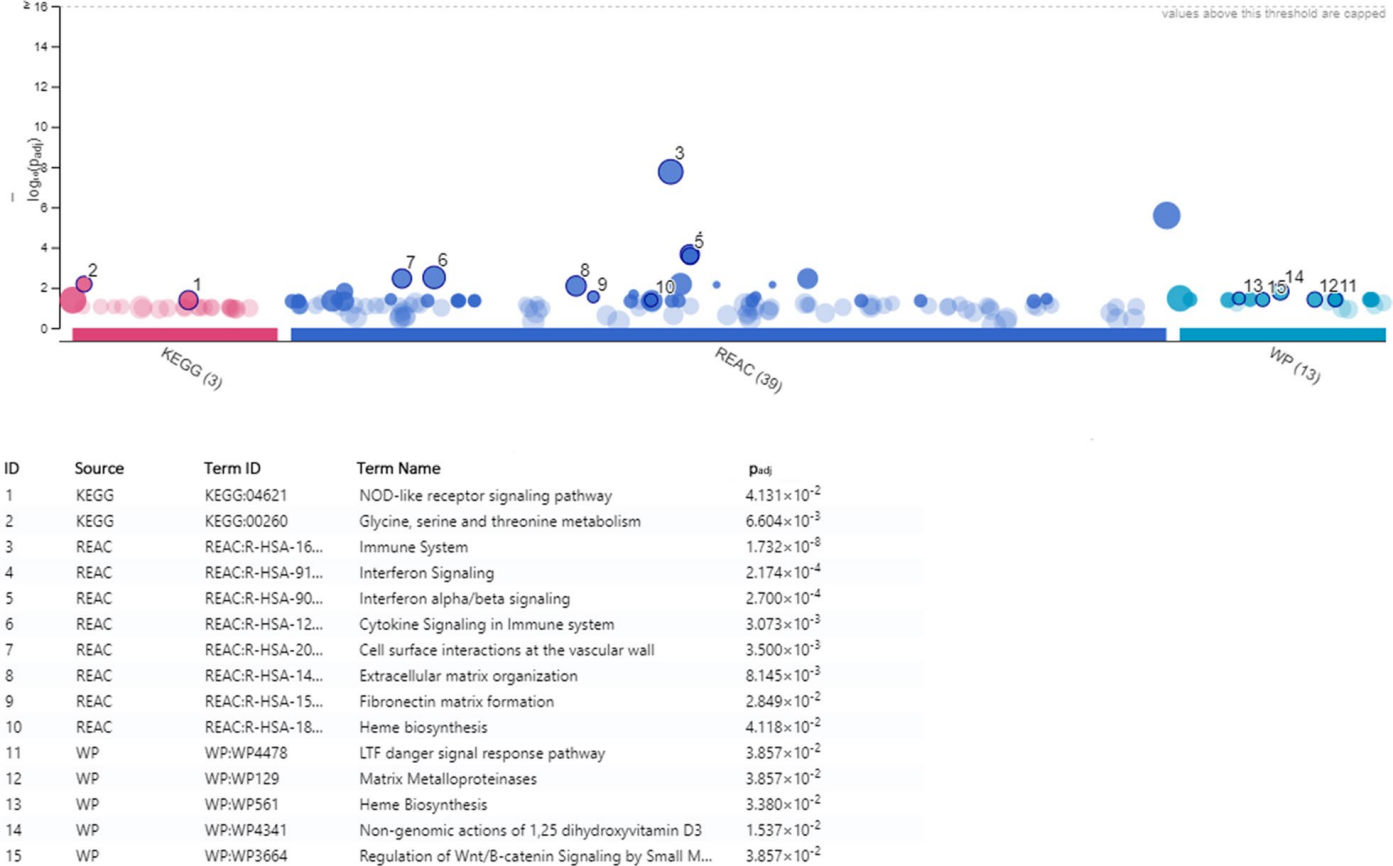
Pro-inflammation and bone metabolism affecting candidate pathways, which are differently expressed in patients with Osteogenesis Imperfecta. Analysis performed with g:GOSt functional profiling tool with Reactome (REACC), WikiPathways (WP) and KEGG annotation sets. Illustrated candidate pathways with adjusted *p* values < 0.05

Thirty-six out of the 39 DEGs were analyzed in the pathway inquiry of g:GOSt tool; three genes (*DEFA1B_DUP3, DEFA1_DUP3, LOC400931*) were not included in the analysis because of an unknown gene annotation in available data sets. A complete list of all statistically significant pathways, identified by the g:GOSt tool is shown in Additional file 2. Reactome, WikiPathways and KEGG analyses identified 39, 13 and 3 altered pathways respectively (Fig. 3).

Reactome annotation revealed an association of 13 DEGs with the immune system (p-value 1.73E−08), supporting the involvement of a pro-inflammation profile in the OI pathological phenotype. Four genes were associated with the interferon (IFN) signaling pathway (*IFI27, RSAD2, IFITM3, GBP7*; *p* value 2.17E−04), with particular involvement of three of them in the IFN-α/β signaling pathway (*IFI27, RSAD2, IFITM3*; *p *value 2.70E−04). Similarly, cytokine signaling in the immune system showed differential expression (*IFI27, RSAD2, IFITM3, SDC1, GBP7*; *p* value 3.07E−03).

In Reactome analysis, besides the immune system pathways, some of the dysregulated networks were connected to ECM functioning: ECM organization (*MMP8, SDC1, CEACAM6;* *p* value 8.14E−03), fibronectin matrix formation (*CEACAM6;* *p* value 2.85E−02) and activation of matrix metalloproteinase (*MMP8;* *p *value 4.851E−02). Lastly, some of the identified pathways were linked to cell surface interactions with the vascular wall (*SDC1, CEACAM6;* *p* value 3.50E−03) and heme biosynthesis (*ALAS2;* *p* value 4.12E−02).

Heme biosynthesis (*ALAS2*; *p* value 3.38E−02) and matrix proteinases (*MMP8*; *p* value 3.86E−02) pathways were also highlighted by WikiPathways analysis. Wikipathways analysis also underlined bone metabolism affecting pathways, like regulation of Wnt/β-catenin signaling by small molecule compounds (*DKK3;* *p* value 3.86E−02), the LTF danger signal response pathway (*LTF*; 3.86E−02) and non-genomic actions of 1.25 dihydroxyvitamin D3 (*RSAD2, IFI44L*; 1.54E−02).

KEGG dataset analysis via g:GOSt tool underlined glycine, serine and threonine metabolism (*ALAS2, GLDC*; *p* value 6.60E−03) and the NOD-like receptor signaling pathway (*DEFA3, GBP7*; *p* value 4.13E−02).

We also used MinePath analysis for the identification of differential pathways and networks. MinePath analysis also utilizes the KEGG pathway repository, which, additionally, in contrast to g:GOSt, aims to identify sub-paths that functionally differentiate between the expression profiles of samples assigned to different phenotypes. The analysis indicated a presence of difference among interactions and interconnections of DEGs between the OI patients and healthy controls. All the identified pathways with p-value < 0.05 (n = 29) are available in Additional file 3.

According to MinePath analysis, numerous altered networks were related to signaling pathways: MAPK (*p* value 5.92E−11), chemokine (*p* value 9.67E−09), Osteoclast differentiation (*p* value 5.38E−05), Ras (*p* value 1.06E−04), Notch (*p* value 8.21E−03), TNF (*p* value 9.90E−03), vascular smooth muscle contraction (*p* value 7.65E−03) and Wnt (*p* value 3.29E−02) (Additional files 4 to 8).

## Discussion

In the current pilot study, we performed a transcriptome analysis of the whole blood in a cohort of OI patients with *COL1A1* splice site and frameshift pathogenic variants and their healthy relatives. We succeeded in identifying differential expression of pro-inflammatory and bone metabolism pathways in blood cells of individuals affected by OI.

The *IFI27* gene—the most significant DEG candidate—is a member of the IFN type I signaling pathway and contributes to apoptosis and signal transduction [6]. The gene is associated with numerous chronic inflammatory states. Up-regulation of the *IFI27* gene was previously reported in psoriasis, diffuse large B-cell lymphoma and fatigued patients after radiation therapy [29–33].

In addition to the *IFI27* gene*, IFITM3*, *RSAD2* and *GBP7* are the other genes involved in IFN signaling and in the other key pathways that are differentially expressed in our Reactome analysis. IFN I family includes IFN-α and -β, which are the cytokines produced by leukocytes and fibroblasts. However, IFN-γ also referred to as IFN type II, is known for its involvement in bone metabolism regulation and is produced by T-lymphocytes [34]. *RSAD2, IFITM3* and *IFI27* are involved in IFN-α/β signaling, while the *GBP7* gene is involved in IFN-γ signaling.

All three IFNs (IFN-α, -β, -γ) are known to decrease collagen synthesis. IFN-α stimulates collagenase activation and inhibition of *COL1A2* gene expression. It is an antagonist of the TGF-β/Smad3 pathway, which promotes bone formation and osteoblast differentiation [35]. In a row with the other cytokine signaling pathways (i.e. Notch, Wnt, fibroblast growth factor), TGF-β/Smad3 pathway controls osteogenesis and bone tissue homeostasis [36]. IFN-β, for its part, inhibits collagen synthesis and activates osteoclastogenesis via IFN-β/STAT1 pathway activation [37]; while IFN-γ is known to downregulate collagen synthesis and to promote bone resorption through osteoclastogenesis via activation of a RANKL/TNFα pathway [38–40].

Our data supports the important role of inflammation in OI pathophysiology; this is in addition to the main OI genetic variant that might alter the bone phenotype of the patients. In addition to IFNs, MAPK, Ras, Notch, TNF and Wnt signaling and osteoclast differentiation pathways are the other significant pathways found in MinePath tool analysis, supporting the alteration of pro-inflammatory pathways and bone homeostasis in patients with *COL1A1* OI variants.

According to our results, one of the upregulated DEGs was the *IFITM3* gene. Interestingly, this gene is closely related to the *IFITM5* gene, which produces a protein of the interferon-inducible trans-membrane (IFITM) family and is known to cause OI type V (*IFITM5*, c.-14C > T) and VI (*IFITM5*, c.119C > T, p.(Ser40Trp)) [41–43]. However, *IFITM3* knockdown did not cause any skeletal phenotype; the gene expression was described to affect MAPK pathway activation and influence TGF-β-Smads-MAPK pathway [44, 45]. Similarly, one of the strongest candidate DEGs was the *RAP1GAP* gene, which encodes an enzyme that binds to the Rep protein and triggers numerous signaling pathways, including the Ras-Raf-MAPK (ERK1/2) cascade [46, 47]. Recently, a connection between OI and alterations in the ERK1/2 pathway was described. A homozygous loss of function variant in the *CCDC134* gene was reported to cause severe OI in three Moroccan patients [48]. The gene inhibits ERK1/2 phosphorylation, causing down-regulation of *COL1A1* and *OPN* in the affected patients. Although the expression of the *CCDC134* gene was not altered in our patients with *COL1A1* variants and mild OI, current data provides a connection between collagen I expression defect in OI and the ERK1/2 pathway. Similarly, the pathway was previously reported to increase bone loss in various pathological conditions, like arthritis and osteoporosis [23, 49].

The lactoferrin protein, a product of the LTF gene, is another DEG with immunomodulatory function and associated with the MARK pathway. It participates in activation of immune cells at inflammation sites [50], and is directly connected to differentiation of osteoblasts, anabolic and anti-apoptotic effects on osteoblasts and regulation of bone growth [51, 52]. Moreover, lactoferrin was reported to inhibit osteoclastogenesis, which is considered to be a potential therapeutic target for bone diseases, including osteoporosis [53, 54]. Up-regulation of the *LTF* gene could represent a rescue mechanism against bone fragility caused by collagen I defects.

One more interesting DEG is the *DKK3* gene. Dickkopf-related protein 3 is involved in bone formation via inhibition of the Wnt signaling pathway [55, 56]. Homozygous *WNT1* pathogenic variants are a cause of OI type XV, whereas heterozygous pathogenic variants and variants in the *LRP5* gene, associated with the Wnt pathway, cause osteoporosis [57, 58]. According to our data, the *DKK3* gene is downregulated in OI patients, which could be speculated as one of the compensatory mechanisms, targeted to avoid inhibition of the Wnt signaling pathway and increase osteogenesis. Indeed, OI bone was described as having a higher turnover and increased number of osteoblasts [7].

Our results share a similarity with the study of Zimmerman et al*.* of osteocyte transcriptome in the OI murine model [59]. Despite the fact that, in contrast to our study, Zimmerman et al*.* used a bone biopsy to explore osteocyte transcriptome, the results of both studies agree on dysregulation of the Wnt and TGF-β pathways’ in collagen-related OI. However, in contrast to the mice study of osteocyte transcriptome, we additionally identified a role of IFN signaling in the whole blood of our human OI patients. We suppose that differences might arise due to greater sensitivity of the blood tissue towards an inflammatory response.

In our view, the transcriptional pattern observed in OI patients not only reflected a pathological state in OI patients, like inflammation, but also revealed compensatory mechanisms of the body, helping to resist abnormal collagen I synthesis and ultimately an effort to modify phenotype. Collagen-related OI mainly alters ECM via decreased collagen I deposition or its abnormal structure. OI bones are described as having a reduced amount of ECM and poor lamellar structure [60]. Reactome and WikiPathways analyses highlighted that both ECM organization and matrix-metalloproteinases pathways were differently expressed in OI patients. One of the DEGs in the current network is the *MMP8* gene that encodes a collagenase, which cleaves the triple-helical structure of types I, II and III collagen. Upregulation of MMP-8 promotes osteoclast activity and differentiation. Interestingly, mice with homozygous mutations in *Col1a1* on MMP8 cleavage sites showed an increased bone, osteocyte and osteoblast deposition [25, 61, 62]. High expression of the *MMP8* gene may point towards a protective mechanism, which increases degradation of an abnormal collagen, synthetized by the mutated *Col1a1.*

We also identified a cross-relation of OI pathophysiology and other collagen-related skeletal diseases. Upregulated expression of the *ADGRG7* gene (*GPR128*) is associated with another rare skeletal dysplasia: Fibrogenesis Imperfecta Ossium. Similar to OI, this disorder is characterized by abnormal collagen, poor bone mineralization, osteopenia/osteoporosis, fractures and bone pain [63, 64]. The gene had the highest upregulated logFC, which could be a reflection of the common pathological state of both the disorders.

Moreover, we identified an altered expression of non-genomic actions of 1,25-dihydroxyvitamin D3 pathway. This finding is particularly interesting because insufficiency of vitamin D is known to be a common problem in OI patients, which may be related to increased bone turnover or bisphosphonate treatment [65]. Interestingly, in OI children vitamin D levels were correlated to bone density, suggesting that this pathway could be one of the modification factors for OI phenotype [66]. However, it should be be noted that similar to OI patients, individuals with osteoporosis also suffer from vitamin D insufficiency, but previous analysis of postmenopausal osteoporosis patients did not reveal any dysregulation of this pathway [23].

The current study is a pilot study with a limited cohort. In order to confirm our findings and to increase its statistical power, a whole blood transcriptome analysis in larger OI cohorts is needed.

The study patients represented relatives from three OI families. Thus, our cohort included pediatric and adult patients of both sexes. Age and gender are known to cause variations in Wnt signaling, as well as MAPK-ERK 1/2 pathways [67, 68]. Additional analysis of these subgroups may reveal new details of bone metabolism variations between pediatric and adult OI populations, as well as male and female OI patients.

## Conclusions

In the current pilot study we explored transcriptome patterns of the whole blood serum in OI patients, with the aim of revealing the molecular pathways involved in OI pathological mechanisms and to explore blood transcriptome potential for profiling a monogenic bone-related disorder, like OI.

Most of the significant DEG candidates were associated with the IFN signaling pathway (*IFI27, IFITM3, RSAD2* and *GBP7*). Additionally, we found that dysregulation of the other cytokine signaling pathways reflects an inflammatory component as a key feature in the blood of OI individuals. Furthermore, our data revealed that significant changes take place in bone metabolism pathways. Upregulation of *MMP8, LTF, ADGRG7* and downregulation of *DKK3* genes reflected a complex effect of a collagen I OI-related variant on the bone structure and homeostasis. Upregulation of the genes connected to vitamin D and associated IFN-α stimulation (*RSAD2, IFI44L*) may reflect consequences of an altered vitamin D metabolism in OI patients, underlining a complex effect of the disorder.

The results of this study are very promising and further peripheral blood transcriptome analysis in bigger cohorts of OI patients is likely to increase the power of the study. Such large-scale studies will help to reveal details about inflammation and bone metabolism signatures in OI patients and will confirm the findings of the current work. Analysis of different OI type subgroups may bring extra benefits for potential identification of the biomarkers of disease severity and novel therapy targets.

## Supplementary information

**Additional file 1**. Differently expressed genes with FDR<0.05 in Osteogenesis Imperfecta patients compared to healthy controls.

**Additional file 2**. Functional profiling of the 39 DEGs according to the web-based g:GOSt tool. The table includes Reactome, KEGG and WikiPathways annotation sets with pathways’ adjusted p-values <0.05.

**Additional file 3**. Significantly dysregulated pathways in OI patients compared to healthy controls according to MinePath tool analysis. The table includes KEGG annotation with p-values <0.05.

**Additional file 4**. Significantly dysregulated MAPK signaling pathway in OI patients compared to healthy controls according to MinePath tool analysis. Green – relations functional in healthy controls, red - relations functional in OI patients.

**Additional file 5**. Significantly dysregulated Osteoclast differentiation pathway in OI patients compared to healthy controls according to MinePath tool analysis. Green – relations functional in healthy controls, red - relations functional in OI patients.

**Additional file 6**. Significantly dysregulated vascular smooth muscle contraction pathway in OI patients compared to healthy controls according to MinePath tool analysis. Green – relations functional in healthy controls, red - relations functional in OI patients.

**Additional file 7**. Significantly dysregulated TNF signaling pathway in OI patients compared to healthy controls according to MinePath tool analysis. Green – relations functional in healthy controls, red - relations functional in OI patients.

**Additional file 8**. Significantly dysregulated Wnt signaling pathway in OI patients compared to healthy controls according to MinePath tool analysis. Green – relations functional in healthy controls, red - relations functional in OI patients.

## Data Availability

The datasets used and analyzed during the current study are available from Gene Expression Omnibus (GEO) repository. Series accession number GSE160207 (https://www.ncbi.nlm.nih.gov/geo/query/acc.cgi?acc=GSE160207).

## Abbreviations

DEGs: Differently expressed genes
DI: Dentinogenesis imperfecta
ECM: Extracellular matrix
FDR: False discovery rate
IFN: Interferon
KEGG: Kyoto Encyclopedia of Genes and Genomes
logFC: Logarithmic fold change
OI: Osteogenesis Imperfecta
RNAseq: RNA sequencing
