# Supplementary figures and images for: RNA sequencing analysis reveals increased expression of interferon signaling genes and dysregulation of bone metabolism affecting pathways in the whole blood of patients with osteogenesis imperfecta
Source: BMC Med Genomics. 2020 Nov 23;13:177. doi: 10.1186/s12920-020-00825-7 (PMC7684725; doi:10.1186/s12920-020-00825-7)

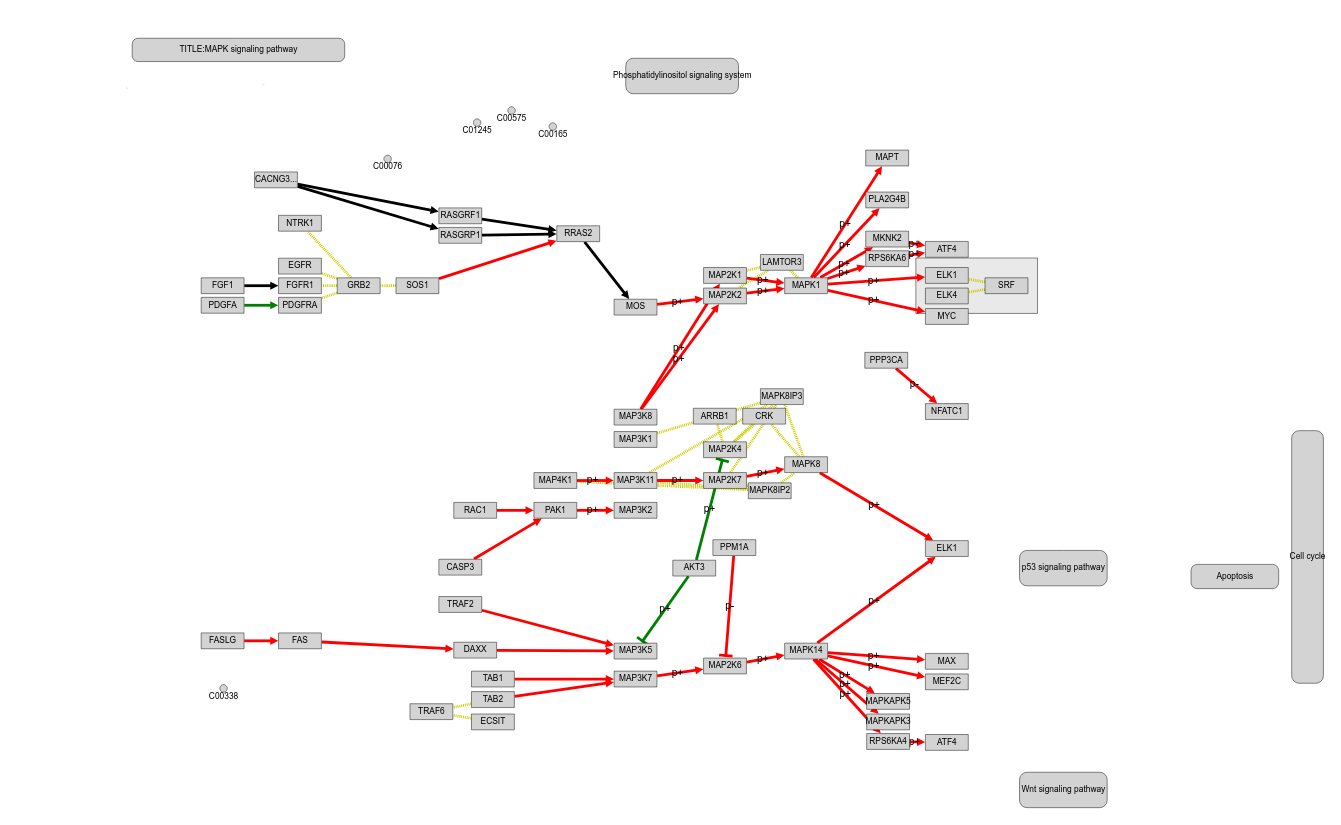

Supplement: Supplementary file 4 — Additional file 4. Significantly dysregulated MAPK signaling pathway in OI patients compared to healthy controls according to MinePath tool analysis. Green – relations functional in healthy controls, red - relations functional in OI patients. [file 12920_2020_825_MOESM4_ESM.png]

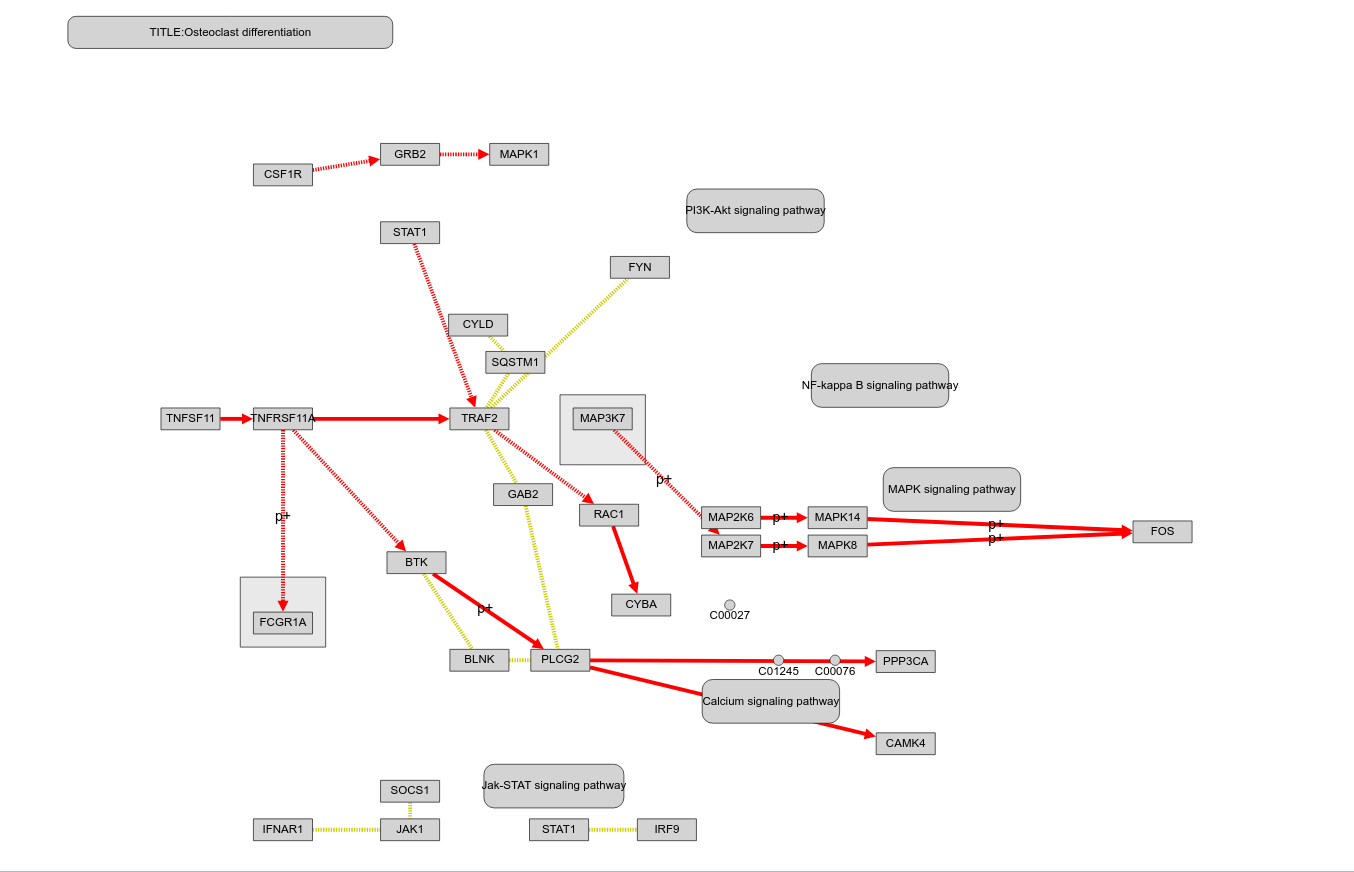

Supplement: Supplementary file 5 — Additional file 5. Significantly dysregulated Osteoclast differentiation pathway in OI patients compared to healthy controls according to MinePath tool analysis. Green – relations functional in healthy controls, red - relations functional in OI patients. [file 12920_2020_825_MOESM5_ESM.png]

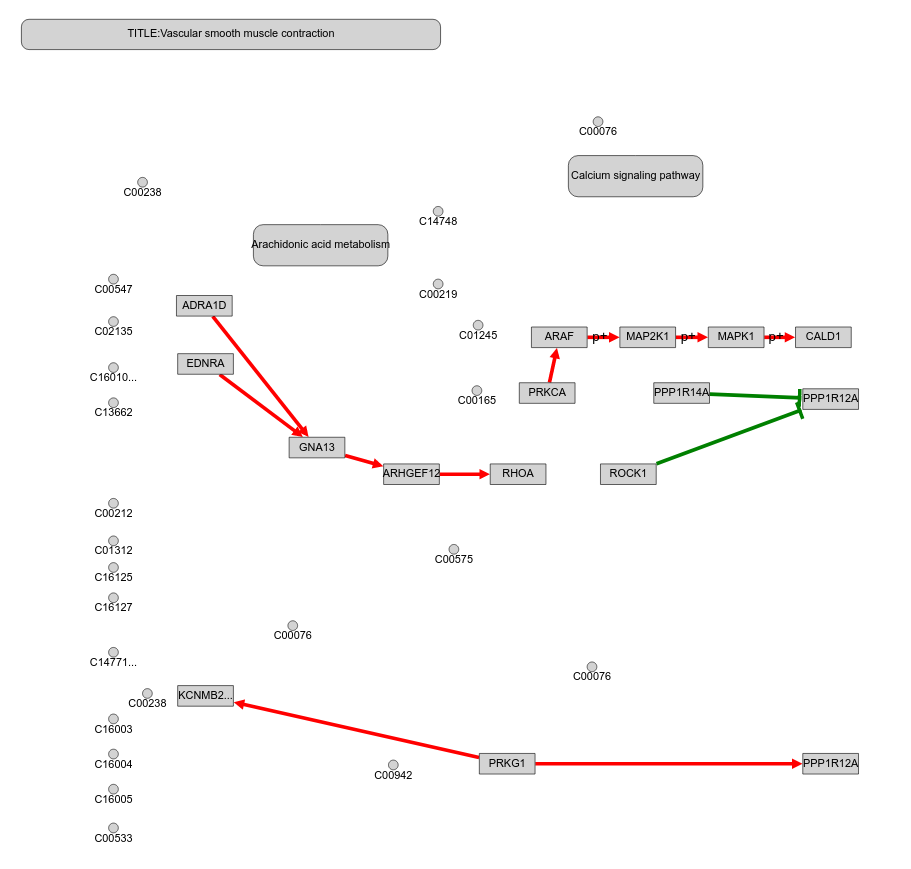

Supplement: Supplementary file 6 — Additional file 6. Significantly dysregulated vascular smooth muscle contraction pathway in OI patients compared to healthy controls according to MinePath tool analysis. Green – relations functional in healthy controls, red - relations functional in OI patients. [file 12920_2020_825_MOESM6_ESM.png]

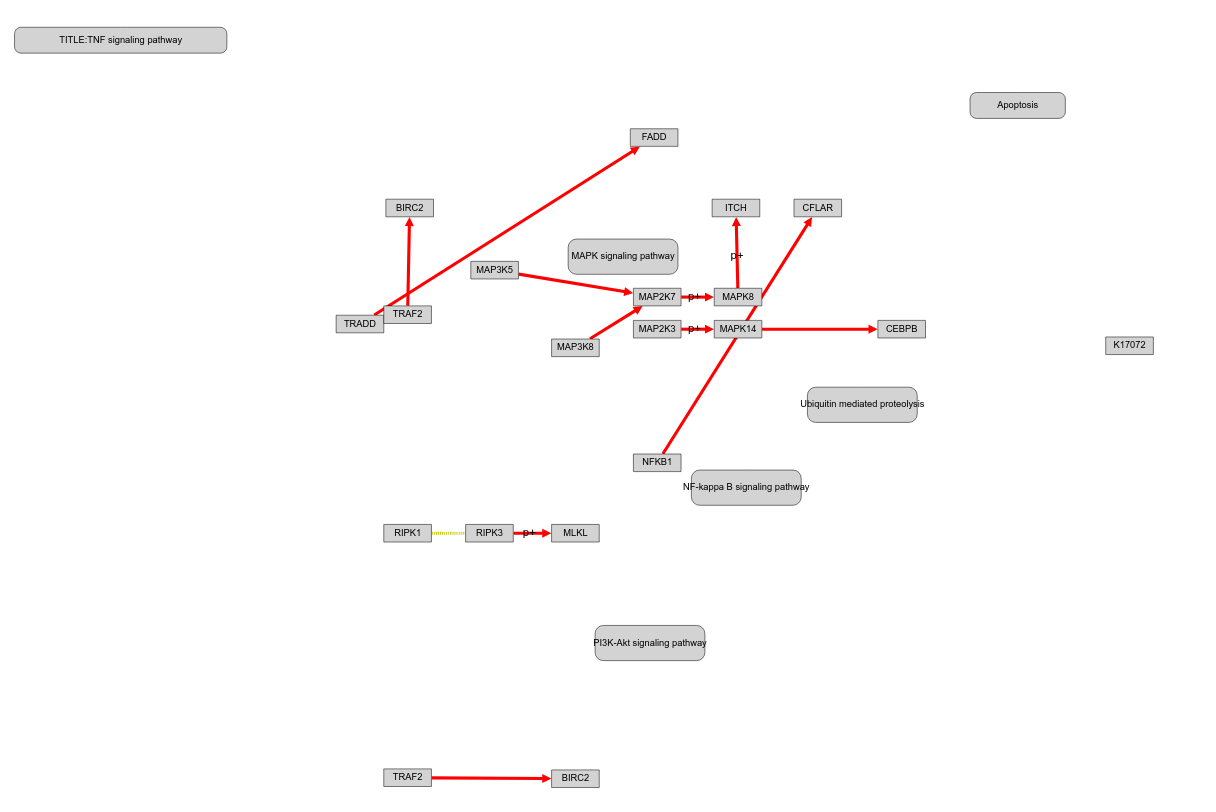

Supplement: Supplementary file 7 — Additional file 7. Significantly dysregulated TNF signaling pathway in OI patients compared to healthy controls according to MinePath tool analysis. Green – relations functional in healthy controls, red - relations functional in OI patients. [file 12920_2020_825_MOESM7_ESM.png]

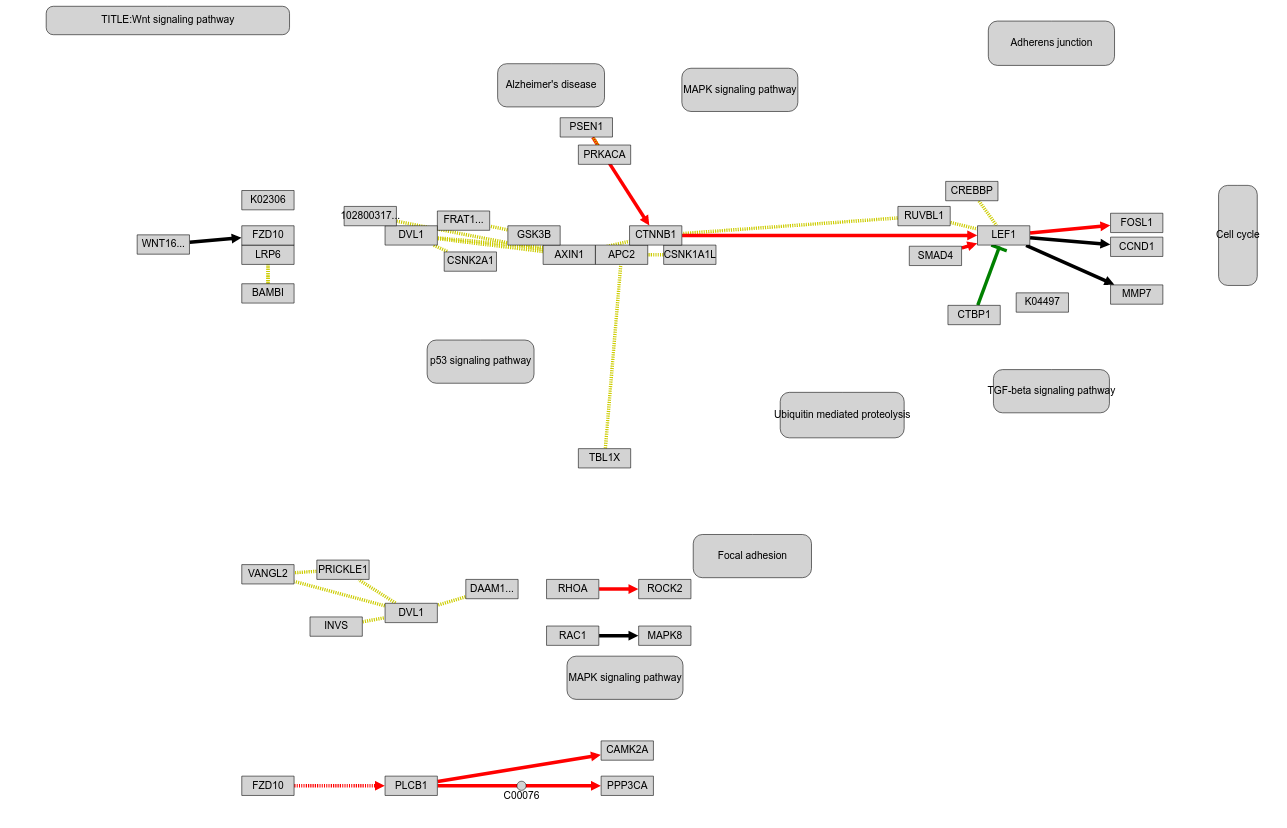

Supplement: Supplementary file 8 — Additional file 8. Significantly dysregulated Wnt signaling pathway in OI patients compared to healthy controls according to MinePath tool analysis. Green – relations functional in healthy controls, red - relations functional in OI patients. [file 12920_2020_825_MOESM8_ESM.png]
